# Validation of the New Lucerne ICF Based Multidisciplinary Observation Scale (LIMOS) for Stroke Patients

**DOI:** 10.1371/journal.pone.0130925

**Published:** 2015-06-25

**Authors:** Beatrice Ottiger, Tim Vanbellingen, Claudia Gabriel, Elisabeth Huberle, Monica Koenig-Bruhin, Tobias Plugshaupt, Stephan Bohlhalter, Thomas Nyffeler

**Affiliations:** Neurology and Neurorehabilitation Center, Luzerner Kantonsspital, Luzern, Switzerland; Fraunhofer Institute for Cell Therapy and Immunology, GERMANY

## Abstract

**Background:**

Valid and multidisciplinary assessment of a stroke patient's ability to perform activities of daily living is very important to define individual goals and to plan targeted rehabilitation. Until today, there is no observation scale that relies on International Classification of Functioning, Disability and Health (ICF). The aim of the present study was to develop and evaluate the reliability and validity of a new multidisciplinary observation scale for stroke patients, shortly called LIMOS, which is based on ICF.

**Methods:**

In a first phase, LIMOS was defined, using a Delphi approach, by an expert panel and a pilot testing was conducted in a small group of stroke patients (n =10) to investigate feasibility and practicability. In a second phase, LIMOS was assessed for its reliability (internal consistency and test-retest reliability) and validity in a large cohort of stroke patients (n = 102). For convergent validity, the correlation between total scores of the LIMOS and the Functional Independence Measure (FIM) was assessed.

**Results:**

LIMOS consisted of seven ICF chapters incorporating 45 domains. A high internal consistency (=0.98) of LIMOS was found. Furthermore, good test-retest reliability at item and subscale level was found. Principal component analysis revealed that among the seven ICF chapters, four components could be found: (1) interpersonal activities, mobility and self-care, (2) communication, (3) knowledge and general tasks, and (4) domestic life. Significant associations were found between LIMOS and the FIM indicating good convergent validity.

**Conclusions:**

The new LIMOS is a reliable and valid observation scale for stroke patients based on ICF, which can be used by a multidisciplinary team working in a neurorehabilitation setting.

## Introduction

An estimated 50 million stroke survivors worldwide currently cope with significant physical, cognitive and emotional deficits. Up to 74% of these survivors still require some assistance or fully depend on the aid of a caregiver when they perform activities of daily living (ADL) such as grooming or walking outside [1– 3]. Several neurological dysfunctions (e.g. motor, sensory, visual) and neuropsychological deficits (memory, attention, language) may explain why stroke patients experience disability in ADL, in severe cases even years after discharge from the rehabilitation center [4].

To optimize functional outcome of a stroke patient, a valid detection of a patient’s ability to perform ADL is unequivocal at the start as well as at the end of rehabilitation procedures. This enables clinicians to set measurable treatment goals, to make appropriate discharge arrangements, or to anticipate the need for community support [3, 5]. To measure disability, clinicians and researchers often use basic ADL measurements, such as the Barthel-Index, the extended Barthel Index, modified Ranking Scale (see for an overview Weimar et al. 2002, [6]) or the Functional Independent Measurement (FIM) [5, 7, 8]. Of all these measurements the FIM, is worldwide one of the most often used measurements. It is a clinician-reported valid measure of disability that has been widely applied for evaluating recovery from stroke. Although FIM has some drawbacks (e.g. ceiling and floor effects; focus on physical domains) and was not primary meant to be comprehensive, it is often used as a gold standard tool to measure ADL [5, 7, 8].

The International Classification of Functioning, Disability and Health (ICF) framework set by the World Health Organization (WHO) was developed to optimize a common language among clinicians [9] and has become a standard in neurorehabilitation. Based on this new classification, ICF core-sets were established for stroke [10, 11]. This facilitated the linking of ICF domains with existing standardized measurements [3, 8, 12, 13]. Recently, new measurements incorporating ICF domains were developed, in the form of self–reported questionnaires [12, 14], or mono disciplinary observation tools for nurses [15], physiotherapists [16] and occupational therapists [17]. The disadvantage of self–reported questionnaires is, however, that they strongly depend on preserved cognitive abilities (i.e. insight) of a stroke patient, possibly biasing the reliability of their answers. Mono disciplinary observation tools, on the other hand, have the disadvantage that they do not reflect the whole condition of a stroke patient, within a multi-disciplinary rehabilitation setting. A valid and reliable comprehensive multidisciplinary observation tool based on ICF for stroke patients is still missing.

The aim of the present study was to develop and evaluate the reliability and validity of a new comprehensive multidisciplinary ICF based observation scale, shortly called LIMOS, in a large cohort of stroke patients. We predicted good test-retest reliability at domain and subscale level of LIMOS. We expected a good internal consistency of the total scale and a homogeneous structure of the different subscales. Furthermore, we hypothesized that the LIMOS will be associated with the FIM, indicating good convergent validity of the scale.

## Materials and Methods

The study was approved by the ethical committee of the state of Lucerne and was consistent with the Declaration of Helsinki, 1975. All patients gave written informed consent prior to participation.

### Phase 1: Development of the LIMOS

#### Development of the main construct of the LIMOS

Experts from the Neurology and Neurorehabilitation Center, Luzerner Kantonsspital, were defined for the development of LIMOS (2 neurologists, 3 nurses, 1 occupational therapist, 1 speech therapist, 3 physiotherapists, and 1 neuropsychologist). This center has a long tradition in using and implementing the ICF since 2002 and already developed an internal simplified ICF manual [18]. Using a Delphi approach, in a consensus round table the chapters (learning and applying knowledge, general tasks and demands, communication, mobility, self-care, domestic life, interpersonal interactions and relationships, major life areas, community, social and civic life) and their incorporating domains (i.e. focusing attention (d160), solving simple problems (d1750), lifting and carrying objects (d430), caring for body parts (d520) etc.) were evaluated.

LIMOS was then presented and discussed within a multidisciplinary workshop. All opinions and remarks were reviewed by the expert panel and further adjustments were done until full agreement was obtained on 9 chapters including 51 domains for the LIMOS.

A new scoring system for the LIMOS was then evaluated. According to the ICF, limitations or restriction in domains are classified as “none”, “slight”, “moderate”, “severe” or “complete” [9]. Regarding the discharge planning after inpatient rehabilitation, it is essential to know how patients perform ADL, i.e. whether they are independent or whether they need further assistance. Therefore, the grade of assistance is a crucial determinant in defining whether someone may be able to return at home or not. Consequently, to better rate limitation or restriction in the clinical setting, we adapted the rating of the ICF domains. A 5 point scale for the LIMOS was thus defined: 1 = patient is not able to fulfil a task or need assistance up to 75% (corresponding to “complete”); 2 = patient is able to fulfil tasks with assistance of 25% to 75% (corresponding to “severe”); 3 = patients is able to fulfil tasks with assistance less than 25% or under supervision (corresponding to “moderate”); 4 = patient is able to fulfil tasks independently but needs more time and/or with auxiliary materials, aids (corresponding to “slight”); 5 = patient is able to fulfil tasks independently (corresponding to “none”).

#### A pilot test of the LIMOS in patients with stroke

A pilot test was conducted in 10 stroke patients to examine the feasibility and practicability of the LIMOS. The responsible neurologists, nurses, physiotherapists, occupational-therapists and speech-therapists of these patients rated their assigned domains of the LIMOS, and these within 72 hours after patient’s admission, and within the last 72 hours before discharge. These members of this pilot phase were not the same as the members of the expert panel. The LIMOS was easy and short to administer, taking only five to ten minutes.

After the pilot test, 7 of the 9 predefined chapters were determined, within an expert panel meeting (members of the development phase), these chapters being: learning and applying knowledge, general tasks and demands, communication, mobility, self-care, domestic life, interpersonal interactions and relationships). The chapters ‘community, social and civic life’ and ‘major life’ were excluded by consensus, since domains such as recreation, religion and political life, within this chapters are not main priority in an inpatient rehabilitation setting.

Based on the review of the expert panel review and the pilot testing, the final construct of the LIMOS was defined, which included 7 chapters incorporating 45 domains (see S1 Table). Based on the 5-point scale (1–5) total score ranges from 45 (lowest) to 225 (highest).

### Phase 2: Examination of the reliability and validity of the LIMOS

In a second phase the reliability and validity of the LIMOS was assessed in a new large group of stroke patients.

One hundred and two in-patients with stroke (33 women, aged between 41–89 years, mean = 69.3, SD = 11.9, time post stroke between 1–69 days, mean = 12.9, SD = 9.7) were prospectively included. Sixty-five patients had an ischemic (A. cerebri media = 43, A. cerebri posterior = 11, A. cerebri anterior = 5) and 37 patients had a hemorrhagic stroke (A. cerebri media = 10, A. cerebri posterior = 1, A. cerebri anterior = 1, others = 25). All patients were admitted to the Neurology and Neurorehabilitation Center, Luzerner Kantonsspital (LUKS), from January to October 2014.

Each stroke patient was assessed with LIMOS and the FIM within 72 hours from admission. The FIM is a standardized assessment for ADL, which includes 18 items rated on a 7-point scale: 1 = total assistance; 2 = maximal assistance; 3 = moderate assistance; 4 = minimal contact assistance; 5 = supervision or set-up; 6 = modified independence; and 7 = complete independence [19]. Total score ranges from 18 (lowest) to 126 (highest). In rehabilitation, the subtotal-summed scores of motor and cognitive subscales (FIM-motor and FIM-cognition) are commonly used to quantify functional independence. The FIM was rated by a multi-discliplinary team.

Descriptive analyses were applied to patients’ demographic variables and behavioral performances (LIMOS, FIM). Internal consistency of the total LIMOS was examined using Cronbach’s alpha. A value above 0.70 is an indicator of a satisfactory homogeneity of items within the total scale. For clinical applications, values above 0.90 are recommended [20].Test-retest reliability of item scores was examined by computing the Kappa of all item scores in a subgroup of 30 patients, which were assessed twice by the same rater (mean delay between test and retest was 2 days; the range was one week). In another group of 15 patients, inter-rater reliability of item scores was assessed by two independent raters. According to Landis and Koch [21] a kappa value between 0.21–0.40 indicates “fair” agreement, between 0.41 and 0.60 “moderate” agreement, between 0.61 and 0.80 “substantial”, and above 0.80 “almost perfect”. Agreement between subscale scores was examined using intra-class correlations (ICC). ICC values should be at least 0.75 [22]. Construct validity was analysed by examining the dimensionality of LIMOS by using a principal component analysis. Convergent validity was assessed by using Pearson’s correlation analyses to assess the association between the LIMOS total scale and the FIM total scale. In addition, associations between subscales of the LIMOS and the subscales FIM mobility and FIM cognition were tested. Scales measuring similar concepts should show correlations, r > 0.60. Furthermore, an association between the LIMOS subscale and the Barthel Index, which was indirectly derived from the motor items of the FIM [23], was tested.

Level of significance was set at p = 0.05 (two-tailed). All values are expressed as mean ± standard deviation (SD). Statistical analyses were performed using PASW for Windows (version 22.0; SPSS, Inc. Chicago, IL).

## Results

The total scores of the LIMOS ranged between 45 and 221 (mean = 144.8, SD = 45.3), and for the total FIM from 18 to 126 (mean = 86.9, SD = 31.4).

An excellent internal consistency of 0.98 for the total LIMOS scale (n = 45 items) was found. Regarding the test-retest reliability at item level, the majority of items had moderate (n = 43) to almost perfect agreement (range of kappa 0.41–0.84). Two items (n = 2) showed fair agreement (0.32–0.37) only, i.e. washing oneself (d510) and coping night (d598). Intra-class correlations for all the subscales were > 0.75 (range of ICC values 0.76–0.0.95). Inter-rater reliability analyses at item level revealed that most items had moderate (n = 33) to almost perfect agreement (range of kappa 0.41–0.92). Twelve items showed fair agreement.

The principal components analysis resulted in 4 components with an eigenvalue above 1.0. A strong first component had an eigenvalue of 28.52 and explained, 55.92% of the variance. A second component had an eigenvalue of 4.60 and explained 9.02% of the variance. A third component had an eigenvalue of 2.90 and explained 5.69% of the variance. A fourth component had an eigenvalue of 1.94 explaining in total a further 3.80% of the variance. Communalities were between 0.53 and 0.96, also indicating substantial common variance. After varimax rotation the 4 components reflected interpersonal activities, mobility and self-care as a first component, communication as a second, knowledge and general tasks as a third, domestic life as a fourth. Internal consistency of these 4 components ranged from 0.95 to 0.97.

A strong significant association between the total LIMOS scale and the total FIM scale was found indicating excellent convergent validity (r = 0.89, p < 0.0001). Furthermore strong significant associations were found between the subscales LIMOS mobility and FIM mobility (r = 0.90, p < 0.0001) and the subscales LIMOS knowledge and FIM cognition (r = 0.81, p < 0.0001). Weaker associations between the other subscales of the LIMOS (self-care, general tasks, domestic life) and the subscales of the FIM were found and ranged between r = 0.36–0.79, p < 0001). In addition a significant association (r = 0.92, p < 0.0001) between the subscale LIMOS mobility and the Barthel Index, which was indirectly derived from the motor items of the FIM [23], was found.

## Discussion

The present study shows that the new Lucerne ICF based Multidisciplinary Observation Scale (LIMOS), which is based on ICF, is a valid and reliable multidisciplinary observation tool for stroke patients in a rehabilitation setting. The construct of the LIMOS was found to be very homogeneous, including four major components. Furthermore, high test-retest reliability at subscale level of LIMOS was found. At domain level, the majority of domains showed moderate *(n = 43)* to almost perfect agreement. The remaining two domains (i.e. washing oneself (d510) and coping night (d598)) showed fair agreement.

We used the FIM as a criterion to examine the convergent validity of the LIMOS. Our results showed a high degree of correlation between the two scales as well as their subscales, indicating that both instruments measure similar constructs. These results confirm our hypothesis and support the convergent validity of LIMOS in stroke patients. Combining the results of a high uni-dimensionality and convergent validity of the LIMOS, the construct validity of the LIMOS is highly supported. Compared with the FIM, mobility and cognitive aspects in ADL can be assessed in a more detailed way by LIMOS. Furthermore, other aspects of daily functioning are also assessed such as self-care, general tasks and domestic life, which may explain the weaker associations found between these subscales and the subscales of the FIM. Consequently, the LIMOS is more comprehensive than the FIM. Having said that, the LIMOS is still easy and short to administer, as each discipline rates their own subpart within the whole scale, requiring only five to ten minutes per discipline. The short administration time of the scale is thus another important advantage of the LIMOS in busy rehabilitation centers. The current trend to shorten the length of hospital stay in rehabilitation settings, and the increasing demand for efficiency in the continuum of stroke care, imply that knowledge about the outcome of ADL is crucial to optimize stroke management. The LIMOS provides a realistic goal-setting and enables early discharge planning.

When developing new outcome scales, it is recommended that these should incorporate the ICF framework, therefore increasing their relevance in clinical practice [24]. Since the LIMOS is based on the ICF, it provides a common understanding and language of functioning, disability, and health used by physicians, nurses, therapists, and other health professionals [4, 25]. The fact that the LIMOS differentiates from basic to complex ADL’s, possible floor and ceiling effects—which have been described for the FIM [5, 26]—can be avoided. Every patient, mildly or severely affected, can be observed and classified with LIMOS. To assess subjective individual goals of a patient, self-reported ICF questionnaires are still needed, and for specific sensory-motor and cognitive dysfunctions, standardized assessments should still be used. Recovery after stroke can be the result of both substitution and restitution within the defined levels of the ICF classification [27]. LIMOS describes the possibilities of a patient in performing activities, irrespective whether recovery involves restitution (e.g. without hand splint) or substitution (e.g. with hand splint). It is therefore essential that in the clinical setting, therapists interpret the LIMOS in the context of compensatory aids.

A limitation of the present study is that LIMOS is not yet validated for other neurological diseases. Regarding test-retest two items showed fair test-retest reliability (i.e. washing oneself (d510) and coping night (d598)). Furthermore, twelve items showed fair inter-rater reliability. All these items we still kept due to their high content validity.

In conclusion, LIMOS is a new valid observation scale based on the ICF, which can be used by a multidisciplinary team working in a neurorehabilitation setting. The LIMOS is a useful and comprehensive tool to assess patients’ ADL functions, and to identify patients’ difficulties in performing ADL tasks. It can be used as a standard tool to plan intervention strategies and to assess functional outcome over a longer period of recovery after stroke.

## Supporting Information

S1 Table The LIMOS. The detailed chapters and domains of the LIMOS are shown. Scoring ranges from 1–5. (1 = patient is not able to fulfil a task or needs assistance up to 75%; 2 = patient is able to fulfil tasks with assistance of 25% to 75%; 3 = patients is able to fulfil tasks with assistance less than 25% or under supervision; 4 = patient is able to fulfil tasks independently but needs more time and/or with auxiliary materials, aids; 5 = patient is able to fulfil tasks independently).
(DOC)
