# Correction: Validation of the New Lucerne ICF Based Multidisciplinary Observation Scale (LIMOS) for Stroke Patients

**DOI:** 10.1371/journal.pone.0134186

**Published:** 2015-07-21

**Authors:** Beatrice Ottiger, Tim Vanbellingen, Claudia Gabriel, Elisabeth Huberle, Monica Koenig-Bruhin, Tobias Pflugshaupt, Stephan Bohlhalter, Thomas Nyffeler

The sixth author’s name is spelled incorrectly. The correct name is: Tobias Pflugshaupt. The correct citation is: Ottiger B, Vanbellingen T, Gabriel C, Huberle E, Koenig-Bruhin M, Pflugshaupt T, et al. (2015) Validation of the New Lucerne ICF Based Multidisciplinary Observation Scale (LIMOS) for Stroke Patients. PLoS ONE 10(6): e0130925. doi:10.1371/journal.pone.0130925
