# Supplementary material for: Validation of the New Lucerne ICF Based Multidisciplinary Observation Scale (LIMOS) for Stroke Patients
Source: PLoS One. 2015 Jun 25;10(6):e0130925. doi: 10.1371/journal.pone.0130925 (PMC4481343; doi:10.1371/journal.pone.0130925)
Supplement: S1 Table — The detailed chapters and domains of the LIMOS are shown. Scoring ranges from 1–5. (1 = patient is not able to fulfil a task or needs assistance up to 75%; 2 = patient is able to fulfil tasks with assistance of 25% to 75%; 3 = patients is able to fulfil tasks with assistance less than 25% or under supervision; 4 = patient is able to fulfil tasks independently but needs more time and/or with auxiliary materials, aids; 5 = patient is able to fulfil tasks independently). (DOC) [file pone.0130925.s001.doc]

| **Chapter** | **Domains** | **Main responsibility** | **Scores** |
| --- | --- | --- | --- |
|  |  |  |  |
| **Chapter 1** | **Learning and applying knowledge** |  |  |
| 1.1 | Acquiring basic skills (d1550) | Occupational Therapy |  |
| 1.2 | Acquiring complex skills (d1551) | Occupational Therapy |  |
| 1.3 | Focusing attention (d160) | Occupational Therapy |  |
| 1.4 | Thinking (d163) | Occupational Therapy |  |
| 1.5 | Solving simple problems (d1750) | Occupational Therapy |  |
| 1.6 | Solving complex problems (d1751) | Occupational Therapy |  |
| 1.7 | Applying knowledge, memories facts (d179) | Occupational Therapy |  |
| 1.8 | Applying knowledge, orientation (d179) | Occupational Therapy |  |
| 1.9 | Applying knowledge, visual and spatial perception (d179 | Occupational Therapy |  |
| 1.10 | Calculation (d172) | Occupational Therapy |  |
| 1.11 | Making simple decision (d177) | Occupational Therapy |  |
| 1.12 | Making complex decision (d177) | Occupational Therapy |  |
|  |  |  |  |
| **Chapter 2** | **General tasks and demands** |  |  |
| 2.1 | Undertaking a simple task (d2100) | Occupational Therapy |  |
| 2.2 | Undertaking a complex task (d2101) | Occupational Therapy |  |
| 2.3 | Carrying out daily routine (d230) | Nurses |  |
|  |  |  |  |
| **Chapter 3** | **Communication** |  |  |
| 3.1 | Communication in general | Nurses |  |
| 3.2 | Oral comprehension (d310, d315) |  |  |
| 3.2.1 | Understanding simple basic needs | Speech Therapists |  |
| 3.2.2 | Understanding simple information | Speech Therapists |  |
| 3.2.3 | Understanding complex information | Speech Therapists |  |
| 3.2.4 | Understanding discussion | Speech Therapists |  |
| 3.3 | Understanding written language (d325) |  |  |
| 3.3.1 | Understanding single words | Speech Therapists |  |
| 3.3.2 | Understanding simple text | Speech Therapists |  |
| 3.3.3 | Understanding long text | Speech Therapists |  |
| 3.3.4 | Understanding complex text | Speech Therapists |  |
| 3.4 | Oral expression (d330) |  |  |
| 3.4.1 | Reply to closed questions | Speech Therapists |  |
| 3.4.2 | Communicate wants and needs | Speech Therapists |  |
| 3.4.3 | Make simple statements | Speech Therapists |  |
| 3.4.4 | Make differentiated statements | Speech Therapists |  |
| 3.5 | Writing (d345) |  |  |
| 3.5.1 | Writing common words | Speech Therapists |  |
| 3.5.2 | Writing short notes and news | Speech Therapists |  |
| 3.5.3. | Writing simple text | Speech Therapists |  |
| 3.5.4 | Writing complex text | Speech Therapists |  |
|  |  |  |  |
| **Chapter 4** | **Mobility** |  |  |
| 4.1 | Maintaining a body position (d415) |  |  |
| 4.1.1 | Maintaining a lying position (d4150) | Physiotherapy |  |
| 4.1.2 | Maintaining a sitting position (d4153) | Physiotherapy |  |
| 4.1.3 | Maintaining a standing position (d4154) | Physiotherapy |  |
| 4.2 | Changing a body position (d410) |  |  |
| 4.2.1 | Lying down (d4100) | Physiotherapy |  |
| 4.2.2 | Sitting (d4103) | Physiotherapy |  |
| 4.2.3 | Standing (d4104) | Physiotherapy |  |
| 4.3 | Transferring oneself (d420) - only for wheel chair drivers |  |  |
| 4.3.1 | Transfer oneself deep from the bed to the wheel chair over the left side (d4208) | Physiotherapy |  |
| 4.3.2 | Transfer oneself deep from the bed to the wheel chair over the right side (d4208) | Physiotherapy |  |
| 4.3.3 | Transfer high (d4208) | Physiotherapy |  |
| 4.4 | Lifting and carrying objects (d430) |  |  |
| 4.4.1 | Lifting and carrying objects in the work area (from the hips to the shoulder area) till 2 kilogramm (d4308) | Physiotherapy |  |
| 4.4.2 | Lifting and carrying objects over the shoulder area (till 2 kilogramm) (d4308) | Physiotherapy |  |
| 4.4.3 | Lifting and carrying objects under the hip area (till 2 kilogramm) (d4308) | Physiotherapy |  |
| 4.5 | Fine hand use (d440) |  |  |
| 4.5.1 | Left hand | Occupational Therapy |  |
| 4.5.2 | Right hand | Occupational Therapy |  |
| 4.5.3 | Bimanuel | Occupational Therapy |  |
| 4.6 | Hand and arm use (d445) |  |  |
| 4.6.1 | Left hand | Occupational Therapy |  |
| 4.6.2 | Right hand | Occupational Therapy |  |
| 4.6.3 | Bimanuel | Occupational Therapy |  |
| 4.7 | Walking short distance (d450) |  |  |
| 4.7.1 | Walking for less than a kilometre, such as walking around in rooms or hallways, within a building or for short distances outside (d4500) | Physiotherapy |  |
| 4.7.2 | Walking on different surfaces and around obstacles (d4502, d4503) | Physiotherapy |  |
| 4.7.3 | Walking up and downhill (d4508) | Physiotherapy |  |
| 4.8 | Walking long distance (d460) (more than 1 kilometer) | Physiotherapy |  |
| 4.9 | Stairs (d455) | Physiotherapy |  |
| 4.10 | Moving around, other specified (d455) | Physiotherapy |  |
| 4.11 | Driving wheel chair (d465) |  |  |
| 4.11.1 | Moving around with the wheel chair on the hospital ward / or at home (d465) | Physiotherapy |  |
| 4.11.2 | Moving around with the wheel chair outside of the hospital / or home (d465) | Physiotherapy |  |
|  |  |  |  |
| **Chapter 5** | **Self-care** |  |  |
| 5.1 | Washing oneself (d510) |  |  |
| 5.1.1 | Washing upper body part (d5100) | Nurse |  |
| 5.1.2 | Washing lower body part (d5100) | Nurse |  |
|  |  |  |  |
| 5.2 | Caring for body parts (d520) | Nurse |  |
| 5.3 | Toileting (d530) |  |  |
| 5.3.1 | Regulation urination (d5300) | Nurse |  |
| 5.3.2 | Regulation defecation (d5301) | Nurse |  |
| 5.3.3 | Regulation menstrual care (d5302) | Nurse |  |
| 5.4 | Dressing (d540) |  |  |
| 5.4.1 | Putting on and taking off clothes upper body (d5400 / d5401) | Nurse |  |
| 5.4.2 | Putting on and taking off clothes lower body (d5400 / d5401) | Nurse |  |
| 5.4.3 | Choosing appropriate clothing (d5404) | Nurse |  |
| 5.5 | Eating (d550) | Nurse |  |
| 5.6 | Drinking (d560) | Nurse |  |
| 5.7 | Looking after one's health (d570) | Nurse |  |
| 5.8 | Coping night (d598) |  |  |
| 5.8.1 | Falling asleep and sleeping through the night (d598) | Nurse |  |
| 5.8.2 | Toileting during the night (d598) | Nurse |  |
| 5.8.3 | Changing body position in bed during the night (d598) | Nurse |  |
|  |  |  |  |
| **Chapter 6** | **Domestic life** |  |  |
| 6.1 | Acquisition of goods and services (d620) | Occupational Therapy |  |
| 6.2 | Preparing simple meals (d6300) | Occupational Therapy |  |
| 6.3 | Preparing complex meals (d6301) | Occupational Therapy |  |
| 6.4 | Doing housework (d640) | Occupational Therapy |  |
| 6.5 | Assisting others (d660) | Occupational Therapy |  |
|  |  |  |  |
| **Chapter 7** | **Interpersonal interactions and relationships** |  |  |
| 7.1 | Interpersonal activities (d710) | Nurse |  |
